# Solvent Action of Boric Acid

**Published:** 1898-09

**Authors:** 


Solvent Action of Boric Acid. It has been found by Berrins (*Rep. de Phar.*) that boric acid increases the solubility in water of thymol, phenol, and salicylic acid. The solubility of the former is doubled, while that of salicylic acid is increased more than eightfold.

